# Impact of list price changes on out-of-pocket costs and adherence in four high-rebate specialty drugs

**DOI:** 10.1371/journal.pone.0280570

**Published:** 2023-01-19

**Authors:** William Bruce Wong, Arpamas Seetasith, Anna Hung, Leah L. Zullig

**Affiliations:** 1 Genentech, Inc., South San Francisco, CA, United States of America; 2 Department of Population Health Sciences, Duke University School of Medicine, Durham, NC, United States of America; 3 Center of Innovation to Accelerate Discovery and Practice Transformation, Durham Veterans Affairs Health Care System, Durham, NC, United States of America; 4 Duke-Margolis Center for Health Policy, Durham, NC, United States of America; The University of Texas MD Anderson Cancer Center, UNITED STATES

## Abstract

**Background:**

Insurers manage the cost of specialty medicines via rebates, however it is unclear if the savings are passed on to patients, and whether reducing rebates may lead to changes in patient out-of-pocket (OOP) costs and medication adherence. This study examined two drug classes to understand the impact of reducing list prices to net prices, via lower-priced national drug codes (NDCs) or authorized generics, on patient OOP costs and adherence.

**Methods:**

This retrospective analysis assessed IQVIA PharMetrics ® Plus adjudicated medical and pharmacy claims for commercially insured patients. Patient OOP costs per prescription and payer drug costs were assessed for evolocumab or alirocumab (proprotein convertase subtilisin/kexin type 9 inhibitors [PCSK9is]) or velpatasvir/sofosbuvir or ledipasvir/sofosbuvir (hepatitis C virus [HCV] medications). For PCSK9is and HCV medications, the original and lower-priced versions were compared. Adherence was estimated based on proportion of days covered (PDC) (PCSK9is) and receipt of full treatment regimen (HCV medications).

**Results:**

In total, 10,640 patients were included (evolocumab, 5,042; alirocumab, 1,438; velpatasvir/sofosbuvir, 2,952; ledipasvir/sofosbuvir,1,208). After list price reductions, mean payer drug costs decreased by over 60%, while patient OOP cost reductions ranged from 14% to 55% (evolocumab: 55%, *p* < 0.01; alirocumab: 51%, *p* < 0.01; velpatasvir/sofosbuvir: 30%, *p* < 0.01; ledipasvir/sofosbuvir: 14%, *p* = 0.03). Patients with coinsurance as the largest contributor to their OOP costs had the largest reductions in OOP costs, ranging from adjusted, mean values of US$135 to US$379 (>60% reductions). Six-month PDC for PCSK9is and proportion receiving full HCV treatment regimen were high with the original versions and did not substantially differ with the new, lower-priced versions.

**Conclusions:**

Reducing list prices to approximate net prices (as a proxy for reducing rebates) resulted in lower patient OOP costs, particularly for those with coinsurance. Our findings suggest that future reduction of rebates may assist in patient affordability, although additional transparency is needed.

## Introduction

The list prices for brand name drugs have been under scrutiny in the US in recent years [1–6]. This is particularly true for the specialty drug market, which often treats complex, rare or orphan diseases. While patients with these difficult-to-treat conditions now have more treatment options, the growth of this market, along with the cost of the drugs, has led to the specialty drug market comprising of approximately half of all drug spend in the US [7]. This has led to many concerns, including the cost to insurers and affordability to patients.

One way insurers are able to manage the cost of specialty drugs is through discounts and rebates, offered by pharmaceutical manufacturers, in exchange for access to their formularies [8]. While savings from rebates may potentially be passed to patients, the complexity and lack of transparency of payments from manufacturer to payer and then on to the patient make such potential savings difficult to quantify. Furthermore, specific prescription benefit elements in the US, such as coinsurance and deductibles, may result in patient out-of-pocket (OOP) costs being unaffected by rebates obtained by insurers. Thus, these complexities in the US pharmaceutical reimbursement system have made it difficult for policymakers to address medication affordability for patients [9].

While rebates may get larger over time due to competition, typically list prices do not decline prior to patent expiry [8, 10, 11]. However, recent price reductions prior to the patent expiry of branded drugs in two drug classes occurred, which provide a unique opportunity to potentially inform policy regarding the reduction of patient OOP costs. The first example concerns two proprotein convertase subtilisin/kexin type 9 inhibitors (PCSK9is) used to treat patients at high risk of cardiovascular mortality. Alirocumab and evolocumab were approved in 2015; [12–14] however, significant barriers to access [15] and scrutiny over value led to the list price of both products being reduced by 60% via the introduction of a new national drug code (NDC). PCSK9i manufacturers argued that traditional rebate discounts offered did not always result in lower costs for patients owing to benefit designs, and that this route of price reduction would lead to greater savings for patients, particularly Medicare beneficiaries with coinsurance [16, 17]. Another example involved two hepatitis C virus (HCV) drug combination products: velpatasvir/sofosbuvir and ledipasvir/sofosbuvir. Here, the manufacturer introduced authorized generics, while indicating that this would potentially reduce patient OOP costs [18]. In both examples, list prices of the new versions of the products were set at prices approaching those already paid by payers owing to varying large rebates and discounts provided (net price), and as a result, rebates were reduced [18–20]. However, it is not known whether reduced rebates would lead to OOP savings and if reductions in patient OOP costs would result in improved outcomes, including adherence to therapy.

Pricing changes in PCSK9is and HCV medications provide a rare opportunity to examine how reducing list prices to approximate net prices (thereby reducing rebates) may potentially have an impact on patient OOP costs and outcomes, further informing policy decision making. Furthermore, little is known about the impact of such changes on commercially insured patients, who comprise approximately half of PCSK9i users [21] and about 30% of individuals receiving HCV medications [22]. This study aims to examine the real-world implications of pricing changes for PCSK9is and HCV medications in a largely commercially insured population to understand the extent to which OOP costs can be reduced and whether potential savings may translate into improved outcomes.

## Methods

### Study design

**Fig 1:**
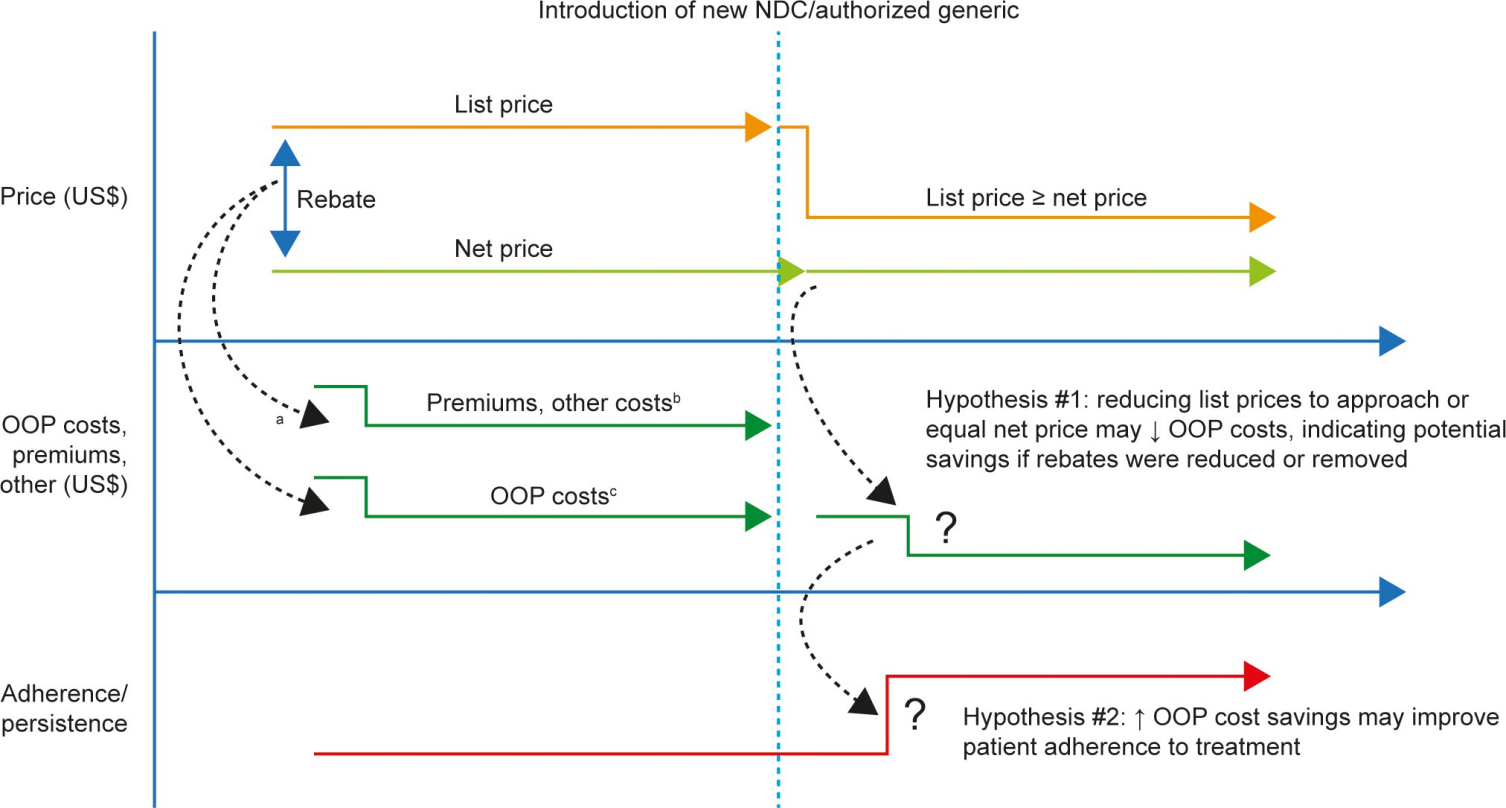
Study concept. ^a^Proportion distributed through different channels is unknown. ^b^Other costs may include items such as administrative fees. ^c^May include copayments, coinsurance, and deductibles. NDC, national drug code; OOP, out of pocket.

This was a retrospective analysis using the IQVIA PharMetrics^®^ Plus database, which comprises of fully adjudicated medical and pharmacy claims data of mostly commercially insured patients. The database contains health plan claims of over 218 million enrollees, 61 million of whom have at least 3 years of continuous enrollment. The PharMetrics Plus data contains patient demographic (year of birth, gender, geographic region, and enrollment information such as enrollment dates, payer type, and health plan type), primary care and specialist visits, hospitalization, pharmacy use, and costs associated with healthcare services. Cost variables included in the database are charge (amount billed for services), allowed (the contracted or accepted reimbursable amount for medications that the health plan agrees to pay service providers such as pharmacies), paid amount (the actual amount paid by health plans to the service providers), coordination of benefit (the reduction in the amount paid to the provider to reflect adjustments as a secondary payer), deductible (the amount required to be paid by patients before health plan will pay for any expenses), copayment (the fixed amount paid by patients for covered services/products after a deductible is met), and coinsurance (a percentage of OOP amount paid by patients for covered services/products after a deductible is met). Data were de-identified according to Health Insurance Portability and Accountability Act patient confidentiality requirements, and institutional review board approval was not required. Data from January 1, 2015 through June 30, 2020 were used. **Fig 1** provides an overview of the study concept.

Four medications which underwent price policy changes, reflected by new NDCs or authorized generics with lower allowed amounts, during the study period were examined: two PCSK9is (evolocumab and alirocumab) and two HCV drug combinations (velpatasvir/sofosbuvir and ledipasvir/sofosbuvir). The list of NDCs used to identify claims can be found in **S1 Table**.

### Selection criteria: PCSK9i

For the PCSK9is, the original NDC was compared with the new NDC (lower priced version). The lower priced version for evolocumab was introduced in October 2018; however, not all formulations were available at the new lower price until January 2019 [23]. The start of the selection window was therefore moved to January 2019 to ensure all drug formulations corresponding to the new NDC were available at the reduced price and to minimize the effect of resetting deductibles at the end of the calendar year [23]. The end of the selection window was May 31, 2019 to allow 6 months of follow-up period for adherence assessment and because the original NDC was phased out toward the end of 2019 and discontinued in 2020 [24].

The selection window for alirocumab was March 4, 2019 (date of introduction of the lower priced version) to December 31, 2019. The date of the first alirocumab or evolocumab claim identified during the selection window was the index date. Given the known access hurdles for PCSK9is, [25] the cohort was restricted to prevalent patients (those with at least one filled prescription prior to the index date) to minimize the effect of interrupted or discontinued use due to prior authorization hurdles during first treatment initiation, and to isolate the effect due to list price change more effectively (rather than other access hurdles).

Inclusion criteria for patients in the PCSK9is included: continuous enrollment in a health plan for at least 12 months before the index date and 6 months after the index date; having at least one filled prescription of PCSK9i prior to index date; no switching of NDCs or drugs during the 6-month post-index period; aged 18 years or older; and having no missing age, payer type, plan type or region of residence data.

### Selection criteria: HCV medicines

For the HCV medications, branded (original) versions of the drugs were compared with the authorized generics (lower priced version). The selection window for velpatasvir/sofosbuvir and ledipasvir/sofosbuvir was the date the authorized generic was introduced, with different NDCs (January 7, 2019 for velpatasvir/sofosbuvir and January 30, 2019 for ledipasvir/sofosbuvir) until March 31, 2020. Similarly, the date of the first claim for velpatasvir/sofosbuvir or ledipasvir/sofosbuvir was defined as an index date.

Inclusion criteria for patients in the HCV medication cohorts included: continuous enrollment in a health plan for 6 months before the index date and 3 months after the index date; no switching of NDCs or drugs during the 3-month post-index period; aged 18 years or older; no HCV treatment before the index date; and having no missing age, payer type, plan type or region of residence data. Additional details on the patient attrition can be found in **S1 Fig.**

### Outcomes

#### OOP costs

We estimated rebates from list price in the quarter prior to introduction of the lower price versions of the drugs using data from SSR Health [26]. These were used to confirm that the new list prices approximated prior net prices and that rebates had declined (**S2 Fig**). Patient OOP costs were calculated as the sum of copayments and coinsurance, excluding deductibles, because list price changes were not anticipated to have an impact on the total deductible required for a given patient (i.e., formulary positioning of the lower priced versions would not change the deductible benefit design). The total amount paid by both the health plan and the patient to the pharmacy for a given medication was estimated based on the allowed amount. If a single OOP cost component (copayment, coinsurance) was missing (seen in <5% of claims in our analysis), OOP costs were calculated as the difference between the allowed amount and the actual amount paid to service providers by the health plan, as well as any coordination of benefits amount. OOP costs were reported as OOP costs per prescription, calculated by dividing patient total OOP costs by the total number of prescriptions filled during follow-up. The driver of OOP costs for each patient was estimated by categorizing individuals based on the largest contributor to their total OOP costs (copayment, coinsurance, unknown or equal). As a sensitivity analysis, OOP costs including deductibles were estimated.

#### Adherence to treatment

Medication adherence was calculated using the proportion of days covered (PDC), defined as the number of days covered with the drug of interest divided by 6 months. PDC was chosen instead of medication possession ratio because it may provide a more accurate estimate in a situation where patients refilled the medication before finishing the current fill of the medication. In such cases, overlapping days supplies were shifted forward [27]. For HCV medications, the US Food and Drug Administration label was used to inform the calculation for patients receiving their full regimen, so the proportion of individuals who received at least three (velpatasvir/sofosbuvir) [28] or two (ledipasvir/sofosbuvir) [29] prescription fills was determined. For ledipasvir/sofosbuvir, two prescription fills defined receipt of a full regimen because treatment-naive patients with genotype 1 and no cirrhosis could be treated for 8 weeks rather than 12 weeks.

### Statistical analysis

Descriptive statistics, including means and proportions, were used to describe baseline patient characteristics, unadjusted PDC values, and HCV drug use, with comparisons made using Chi-squared tests for categorical variables and Wilcoxon two-sample tests with *t* approximation for continuous variables.

Adjusted analyses were performed for OOP costs per prescription for PCSK9is and HCV medications, mean PDC for PCSK9is, and completion of the full regimen for HCV medications. Mean OOP costs per prescription and mean allowed amounts were estimated using a log-link gamma generalized linear model adjusted for age, sex, region, payer type, plan type, polypharmacy (defined as three or more medications with medication supplies overlapping the index date), Charlson Comorbidity Index and index month for all medications in the study. The log-link gamma generalized linear model was selected to provide more precise estimates of the population means as it accounts for high positive skewness commonly observed in healthcare cost [30].

Mean PDC for PCSK9is was also estimated using a generalized linear model with log-link and gamma distribution to address the distribution of PDC values due to truncation. Adjustment variables for both models (lower priced version versus original NDC) were based on results from a previous study examining factors associated with PCSK9i treatment interruption [31] and included age, sex, region, plan type, polypharmacy, index month, PDC in the prior 12 months, time from first ever PCSK9i use prior to the index prescription, prior number of all-cause hospitalizations, baseline chronic kidney disease, baseline use of statins, baseline use of ezetimibe and baseline diabetes in the prior 12 months. Baseline PDC was calculated as the number of days covered with the medication from the first PCSK9i prescription filled (in the 12 months prior to the index date) up to the index date, divided by the number of days between that first prescription filled and the index date. The odds of receipt of a complete HCV regimen were estimated using a logistic regression model adjusted for age, sex, region, payer type, plan type, polypharmacy, Charlson Comorbidity Index and index quarter.

All analyses were performed using SAS Studio Release 3.7 (Enterprise Edition), 2012–2017 (SAS Institute, Inc., Cary, NC).

## Results

### Patient characteristics

In total, 10,640 patients were included in the analysis (evolocumab, 5,042; alirocumab, 1,438; velpatasvir/sofosbuvir, 2,952; ledipasvir/sofosbuvir, 1,208). Patient characteristics are shown in **Table 1**. Across all treatments, age, sex and the number of comorbidities were similar. Among the PCSK9is, baseline mean PDC was statistically significantly different between the original and the lower priced version (*p* < 0.001 for alirocumab and *p* = 0.04 for evolocumab); however, values were numerically similar (original NDC versus lower priced version: 0.85 versus 0.84 for evolocumab, and 0.85 versus 0.83 for alirocumab). Although the mean number of months from first ever PCSK9i use (prior to the index PCSK9i) was similar for the lower priced version and original NDC for evolocumab (*p* > 0.05), the time was significantly longer for the lower priced version of alirocumab than for the original NDC (20.5 versus 16.8 months, *p* < 0.01).

**Table 1:** Baseline characteristics of patients included in the study^a^.

Characteristic	PCSK9is						HCVs					
	Evolocumab			Alirocumab			Velpatasvir/sofosbuvir			Ledipasvir/sofosbuvir		
	Original NDC	Lower Priced Version	*p* value	Original NDC	Lower Priced Version	*p* value	Original NDC	Lower Priced Version	*p* value	Original NDC	Lower Priced Version	*p* value
Total	2,331 (100.0)	2,711 (100.0)		987 (100.0)	451 (100.0)		1791 (100.0)	1,161 (100.0)		827 (100.0)	381 (100.0)	
Mean (SD) age, years	60.8 (9.1)	60.4 (8.7)	0.03	59.2 (8.7)	60.4 (8.8)	0.04	52.3 (12.8)	51.7 (12.9)	0.32	50.9 (13.8)	49.6 (13.5)	0.07
Female	820 (35.2)	938 (34.6)	0.68	358 (36.3)	174 (38.6)	0.40	585 (32.7)	417 (35.9)	0.07	310 (37.5)	149 (39.1)	0.59
Region												
Northeast	353 (15.1)	230 (8.5)	< 0.01	205 (20.8)	70 (15.5)	0.00	198 (11.1)	98 (8.4)	0.08	223 (27.0)	34 (8.9)	< 0.01
South	959 (41.1)	1,542 (56.9)		452 (45.8)	214 (47.5)		1,022 (57.1)	661 (56.9)		437 (52.8)	279 (73.2)	
Midwest	581 (24.9)	669 (24.7)		222 (22.5)	92 (20.4)		412 (23.0)	283 (24.4)		100 (12.1)	46 (12.1)	
West	438 (18.8)	270 (10.0)		108 (10.9)	75 (16.6)		159 (8.9)	119 (10.2)		67 (8.1)	22 (5.8)	
Payer												
Fully insured	1,508 (64.7)	1,704 (62.9)	< 0.01	618 (62.6)	299 (66.3)	0.00	1,061 (59.2)	823 (70.9)	< 0.01	517 (62.5)	293 (76.9)	< 0.01
Self-insured	557 (23.9)	800 (29.5)		325 (32.9)	115 (25.5)		489 (27.3)	241 (20.8)		243 (29.4)	66 (17.3)	
Government	266 (11.4)	207 (7.6)		44 (4.5)	37 (8.2)		241 (13.5)	97 (8.4)		67 (8.1)	22 (5.8)	
Plan type												
HMO	266 (11.4)	278 (10.3)		134 (13.6)	59 (13.1)		376 (21.0)	256 (22.0)		200 (24.2)	56 (14.7)	
PPO	1,845 (79.2)	2,232 (82.3)	0.01	782 (79.2)	349 (77.4)	0.31	1,336 (74.6)	810 (69.8)	< 0.01	594 (71.8)	269 (70.6)	< 0.01
Other	220 (9.4)	201 (7.4)		71 (7.2)	43 (9.5)		79 (4.4)	95 (8.2)		33 (4.0)	56 (14.7)	
CCI category												
0	1,323 (56.8)	1,619 (59.7)	0.26	591 (59.9)	280 (62.1)	0.41	257 (14.3)	158 (13.6)	0.82	130 (15.7)	63 (16.5)	0.79
1	390 (16.7)	406 (15.0)		163 (16.5)	73 (16.2)		28 (1.6)	21 (1.8)		18 (2.2)	6 (1.6)	
2	339 (14.5)	383 (14.1)		146 (14.8)	64 (14.2)		1,008 (56.3)	667 (57.5)		494 (59.7)	223 (58.5)	
3	143 (6.1)	149 (5.5)		49 (5.0)	13 (2.9)		200 (11.2)	117 (10.1)		84 (10.2)	35 (9.2)	
4+	136 (5.8)	154 (5.7)		38 (3.9)	21 (4.7)		298 (16.6)	198 (17.1)		101 (12.2)	54 (14.2)	
Polypharmacy (3+)												
No	386 (16.6)	452 (16.7)	0.89	161 (16.3)	72 (16.0)	0.87	846 (47.2)	567 (48.8)	0.40	434 (52.5)	192 (50.4)	0.50
Yes	1,945 (83.4)	2,259 (83.3)		826 (83.7)	379 (84.0)		945 (52.8)	594 (51.2)		393 (47.5)	189 (49.6)	
DM												
No	1,689 (72.5)	2,055 (75.8)		774 (78.4)	346 (76.7)		NA	NA		NA	NA	
Yes	642 (27.5)	656 (24.2)	0.01	213 (21.6)	105 (23.3)	0.27	NA	NA		NA	NA	
CKD												
No	2,096 (89.9)	2,501 (92.3)	0.00	916 (92.8)	411 (91.1)	0.27	NA	NA		NA	NA	
Yes	235 (10.1)	210 (7.8)		71 (7.2)	40 (8.9)		NA	NA		NA	NA	
No. of prior hospitalizations at baseline, mean (SD)	0.18 (0.6)	0.2 (0.6)	0.13	0.17 (0.5)	0.14 (0.4)	0.49	NA	NA		NA	NA	
Baseline statin use	1,040 (44.6)	1,242 (45.8)	0.40	447 (45.3)	188 (41.7)	0.20	NA	NA		NA	NA	
Baseline ezetimibe use	508 (21.8)	589 (21.7)	0.95	242 (24.5)	89 (19.7)	0.05	NA	NA		NA	NA	
Baseline statin or ezetimibe use	1,235 (53.0)	1,478 (54.5)	0.28	540 (54.7)	230 (51.0)	0.19	NA	NA		NA	NA	
Time from first PCSK9i use, mean (SD), months	13.93 (10.1)	13.60 (10.3)	0.11	16.83 (11.7)	20.53 (13.1)	< 0.01	NA	NA		NA	NA	
Baseline PDC, mean (SD)	0.85 (0.2)	0.84 (0.2)	0.04	0.85 (0.2)	0.83 (0.2)	0.00	NA	NA		NA	NA	
Baseline PDC ≥ 80%	1,706 (73.2)	1,927 (71.1)	0.09	743 (75.3)	317 (70.3)	0.05	NA	NA		NA	NA	

^***a***^All values are n (%) unless otherwise specified.

CCI, Charlson Comorbidity Index; CKD, chronic kidney disease; DM, diabetes mellitus; HCV, hepatitis C virus; HMO, health maintenance organization; NA, not applicable; NDC, national drug code; PCSK9i, proprotein convertase subtilisin/kexin type 9 inhibitor; PDC, proportion of days covered; PPO, preferred provider organization; SD, standard deviation.

### Patient OOP costs

**Fig 2:**
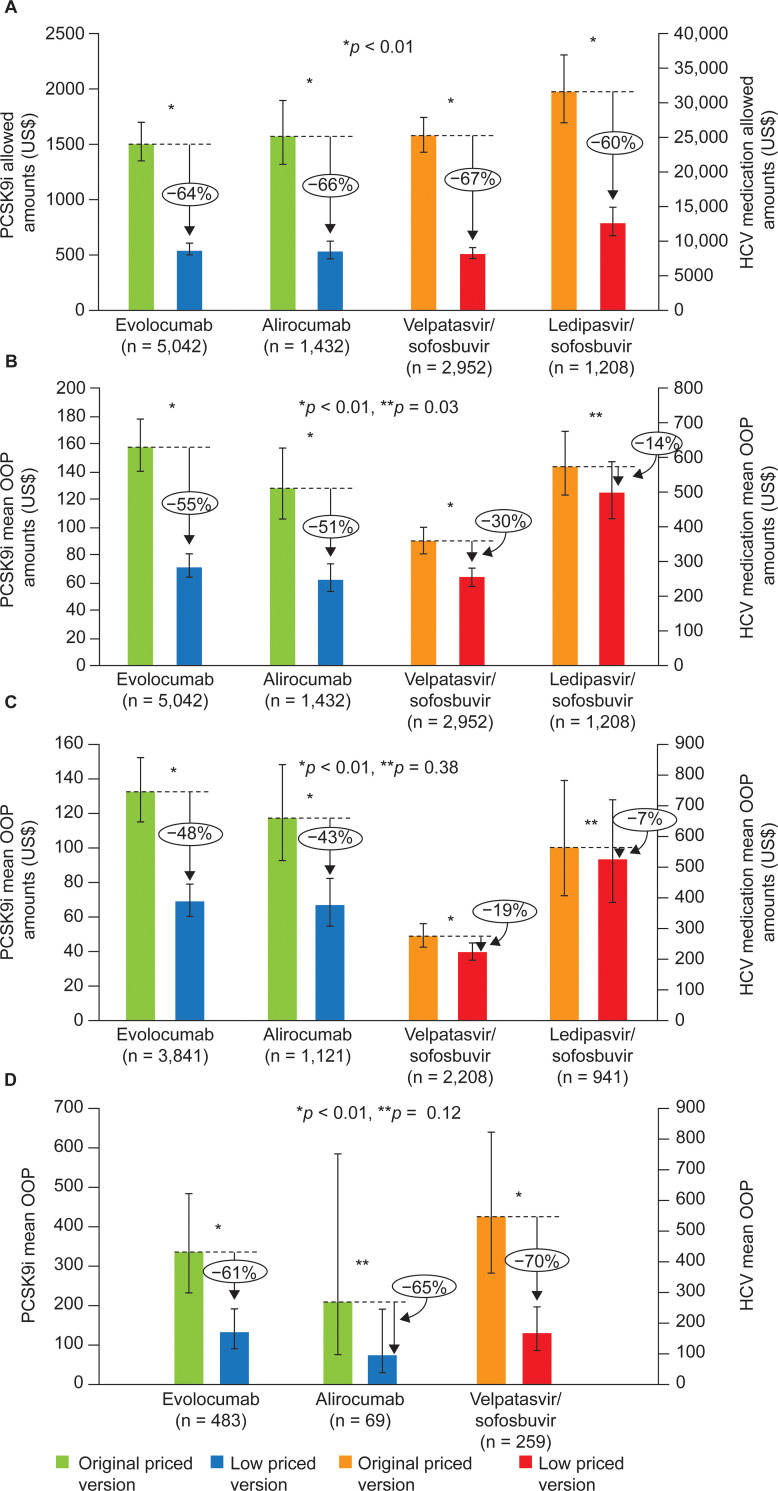
A) Adjusted mean allowed amounts per-prescription. B) Adjusted mean patient OOP per-prescription amounts. C) Adjusted mean OOP: subgroup with copayment as largest contributor to OOP cost. D)^a^ Adjusted mean OOP: subgroup with coinsurance as largest contributor to OOP cost. Model adjusted for age, sex, region, payer type, plan type, polypharmacy (3+ medications), Charlson Comorbidity Index and index month. Allowed amounts are the contracted or accepted reimbursable amount for medications that the health plan agrees to pay service providers such as pharmacies. ^a^Insufficient sample to obtain robust estimates for Ledipasvir/sofosbuvir given data outliers. HCV, hepatitis C virus; OOP, out of pocket; PCSK9i, proprotein convertase subtilisin/kexin type 9 inhibitor.

In adjusted analyses (**Fig 2A**), mean allowed amounts were reduced by more than 60% with the lower priced version for all treatments (reduction of approximately US$1,000 per prescription for PCSK9is, and US$17,000 to US$19,000 per prescription for HCV medications). Percentage and absolute reductions in mean OOP costs per prescription (**Fig 2B**) were less than those observed in allowed amounts (all >60%), with greater percentage reductions (absolute difference [95% confidence interval (CI)], % difference) for the lower priced versions of the PCSK9i (evolocumab: US$87 [US$82–91], 55%, *p* < 0.01; alirocumab: US$66 [US$54–77], 51%, *p* < 0.01) than for the HCV lower priced versions (velpatasvir/sofosbuvir: US$107 [US$86–127], 30%, *p* < 0.01; ledipasvir/sofosbuvir: US$79 [US$7–142], 14%, *p* = 0.03).

Copayment was the largest contributor to OOP costs in the majority of patients (evolocumab: 76% of patients; alirocumab: 78%; velpatasvir/sofosbuvir: 67%; ledipasvir/sofosbuvir: 71%), while coinsurance was the largest contributor of OOP costs in less than 10% of patients (evolocumab: 9.6%; alirocumab: 4.8%; velpatasvir/sofosbuvir: 8.8%; ledipasvir/sofosbuvir: 5.5%). Absolute differences in adjusted mean OOP costs between the original and lower priced versions for those with copayments as the largest contributor to OOP costs were less than US$65, with percentage reductions ranging from 48% to 7% (**Fig 2C**). In contrast, in the subgroup with coinsurance as the largest proportion of OOP costs, absolute mean adjusted OOP costs differences between the original and the new lower priced versions ranged from US$135 to US$379, with percentage reductions over 60% (**Fig 2D**).

**Fig 3:**
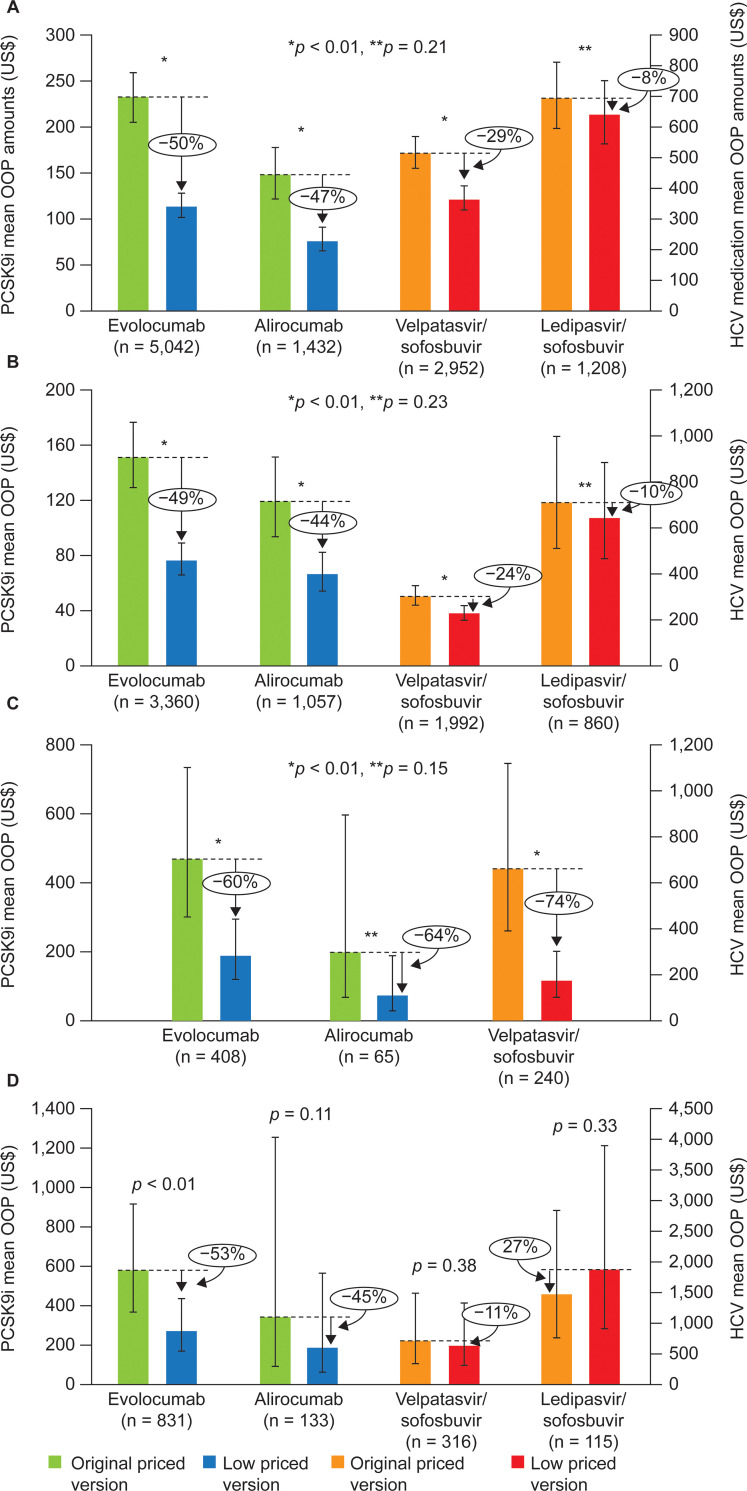
Adjusted mean OOP costs with deductibles included for A) total OOP costs; B) subgroup with copayment as largest contributor to OOP cost; C)^a^ subgroup with coinsurance as largest contributor to OOP cost; and D) subgroup with deductible as the largest contributor to OOP cost. Model adjusted for age, sex, region, payer type, plan type, polypharmacy (3+ medications), Charlson Comorbidity Index, and index month. ^a^Insufficient sample for estimates for Ledipasvir/sofosbuvir. HCV, hepatitis C virus; OOP, out of pocket; PCSK9i, proprotein convertase subtilisin/kexin type 9 inhibitor.

Percentage changes in adjusted mean OOP costs when OOP costs included deductibles were similar to when these costs were not included, although slightly attenuated (**Fig 3A**). When including deductibles in the OOP costs, the subgroup of patients for whom deductibles were the largest contributor accounted for approximately 10–15% of patients (evolocumab: 16.5%; alirocumab: 9.2%; velpatasvir/sofosbuvir: 10.7%; ledipasvir/sofosbuvir: 9.5%). For those with deductibles as the largest contributor to OOP costs, absolute differences in adjusted mean OOP costs between the original PCSK9i NDC and lower priced version differed from that of the original HCV medicines compared with their lower priced versions (**Fig 3B–3D**). Among the subgroup with deductibles as the largest contributor to OOP costs, the absolute differences between the original NDC and the new lower priced version for the PCSK9i (US$155–305) were larger than the subgroup with copayments, as the largest contributor (US$52–74) and similar in magnitude to the coinsurance subgroup (US$124–279). Conversely, the absolute differences between the original and lower priced versions for the HCV medicines (among those with deductibles as the largest contributor) was not significantly different (*p* > 0.05), and in fact more similar to that of the subgroup with copayments as the largest contributor to OOP costs (US$70–73), and smaller in magnitude compared with the coinsurance as the largest contributor to OOP costs subgroup (velpatasvir/sofosbuvir: US$483).

### Adherence and utilization

After adjusting for patient characteristics (**Table 2**), the mean 6-month PDC for the lower priced version of evolocumab was statistically significantly higher than that of the original, but values were numerically similar (mean PDC [95% CI]: 0.82 [0.79–0.85] versus 0.79 [0.76–0.82], *p* < 0.01). Mean 6-month PDCs for the lower priced version compared with the original for alirocumab were similar (mean PDC [95% CI]: 0.82 [0.78–0.87] versus 0.79 [0.74–0.85], *p* = 0.22).

**Table 2:** Adjusted adherence and utilization for the treatments.

Outcome	Drug	Comparison	Mean PDC^a^ (95% CI)	*p* value
6-month adherence	Evolocumab	Lower Priced Version	0.82 (0.79–0.85)	< 0.01
		Original NDC	0.79 (0.76–0.82)	
	Alirocumab	Lower Priced Version	0.82 (0.78–0.87)	0.22
		Original NDC	0.79 (0.74–0.85)	
Outcome	Drug	Comparison	OR (95% CI)^b^	*p* value
At least three prescriptions	Velpatasvir/sofosbuvir	Lower Priced Version vs Original (ref.)	0.79 (0.61–1.02)	0.07
At least two prescriptions	Ledipasvir/sofosbuvir	Lower Priced Version vs Original (ref.)	0.73 (0.39–1.37)	0.33

^a^Adjusted for age, sex, region, payer type, plan type, polypharmacy, Charlson Comorbidity Index, index month, PDC in prior 12 months, time from first proprotein convertase subtilisin/kexin type 9 inhibitor use, prior number of hospitalizations, baseline chronic kidney disease, baseline use of statins, baseline use of ezetimibe and baseline diabetes mellitus, using a log-link gamma generalized linear model.

^b^The odds of receipt of a complete hepatitis C virus medication regimen were estimated using a logistic regression model adjusted for age, sex, region, payer type, plan type, polypharmacy, Charlson Comorbidity Index and index quarter.CI, confidence interval; NDC, national drug code; OR, odds ratio; PDC, proportion of days covered.

The proportion of patients completing their original NDC HCV medication full regimen was 92% and 96% for velpatasvir/sofosbuvir and ledipasvir/sofosbuvir, respectively (see **S2 Table**). After adjusting for patient characteristics (**Table 2**), the odds (odds ratio [OR] [95% CI]) of completing the HCV regimen were not significantly greater with the lower priced version velpatasvir/sofosbuvir (0.79 [0.61–1.02], *p* = 0.07) or ledipasvir/sofosbuvir (0.73 [0.39–1.37], *p* = 0.33) than with the original NDC.

## Discussion

Despite much debate on how to reduce patient OOP costs, there is limited real-world evidence to inform policy discussions. We leveraged real-world data to examine the effect of two price reduction mechanisms in heavily rebated markets on patient OOP costs and adherence to treatment. To the best of our knowledge, this is the first study to examine the effect of reducing list prices to close to that of net prices, providing insight into the complex flow of funds from manufacturer to patients. Our results have implications for two important considerations for policy makers: rebates and cost sharing benefit designs.

In both these examples, the manufacturers chose to introduce new versions of their products while maintaining access to the existing products. In the case of the PCSK9is, dual products were available for a period to allow a smooth transition by minimizing supply chain disruption and allowing insurers to modify existing contracts [16]. In the case of the HCV medicines, the manufacturer provided insurers a choice on whether to cover the branded version or the authorized generic [32]. In both cases, while some payers transitioned patients to the new lower priced versions others continued their patients on the existing higher priced version [33–36]. This was true in our study as well, with three out of the four drugs having more patients in the higher priced version cohort than the lower priced version. One possibility for this observation would be due to the large rebates being offered for the higher priced version of the product (leading to the significant gap between the list price and net price of the products) which may have disincentivized payers to switch to products which may have a lower list price but would result in the loss of funds from the rebates. This slow transition to the new lower price versions may have resulted in potentially avoidable higher OOP costs for patients who remained on the higher price versions during this period.

The reduction of list prices to net prices may be a proxy for potentially reducing rebates, given the difference between the list and net prices are a result of the discounts manufacturers provide to payers. Results of recent studies suggest that the reduction of rebates may provide additional savings to patients, particularly for individuals with coinsurance [37, 38]. Our findings are consistent with these, in that introducing lower list price versions similar to that of the net prices of the original product resulted in lower patient OOP costs in a largely commercially insured population. Savings could also be passed on to patients by other routes, such as monthly premiums; however, this may result in reverse insurance, with the sick subsidizing the healthy, rather than the savings being directed to individuals in need. Our results therefore suggest a minimum level of cost savings that patients might expect to see as a result of reducing list prices to net prices. While there may be opportunities for further OOP cost savings, greater transparency is needed to allow further research into the flow of savings from rebates to the various options available.

While this study may provide some insight into how potentially reducing rebates via new list prices may lower OOP costs, real-world evidence has shown that this may not come to fruition owing to various prescription benefit designs. An analysis of Medicare claims data found that, despite list price reductions of PCSK9is, many Medicare beneficiaries may still face substantial OOP costs owing to shifting of placement from specialty tiers, for which coinsurance is capped between 25% and 33%, to non-preferred tiers, in which cost sharing may be as high as 50% [39]. In the present study, however, a large proportion of the savings in OOP costs was found to be driven by coinsurance, although this involved a minority (5% to 10%) of patients. Nonetheless, the lower OOP costs seen for individuals with coinsurance is consistent with the manufacturers’ expectations and stated rationale for their price reduction approaches [8, 40]. However, given the variation in findings between the Medicare and the commercial analyses, future research is needed to understand the accompanying formulary changes that might occur in reaction to lower list prices or reducing rebates, to inform policy makers on the peripheral implications of policies that target these areas.

Our results suggest that high deductible plans may also be a barrier to reducing the OOP costs for some patients. The reduction in OOP costs among those with large deductibles was observed for the evolocumab but not the HCV medicines (while alirocumab was not statically significant, the magnitude of the effect was larger and the sample size was small), suggesting that patients with high deductible plans may not benefit from reduction of rebates for higher cost drugs. This may be due to the fact that the cost of the HCV medications were likely well over most patient deductible amounts while those patients using PCSK9i whom only filled a single or two prescriptions may still be under their deductible amount. While those utilizing PCSK9i used less of their deductible on the cost of the drug at the new lower priced NDC, it is unknown whether they would still use their full deductible amount owing to the use of other medical services. Additional research is needed to further understand if OOP costs due to overall utilization of healthcare services would differ, despite using less of the deductible on drug costs.

Previous studies have demonstrated that small changes in patient OOP costs may result in substantial changes in adherence to therapy. A prior study of medication adherence in veterans at US Veterans Affairs Medical Centers found that even a US$5 copayment increase (from US$2 to US$7) led to poorer treatment adherence for diabetes, hypertension and hyperglycemic medications [41]. Among a majority commercial population (55% to 85%), US$51 to US$75 appeared to be the threshold for diabetes OOP medication costs at which treatment adherence reduced significantly [42]. Despite observing differences in OOP costs of US$66 to US$107 per prescription with the new list prices, we found little evidence that these differences in OOP costs increased adherence to treatment. This was not surprising given that the mean baseline level of medication adherence for PCSK9is was high (>80%) and that the likelihood of receiving the full HCV drug regimen with the original NDC medication was also high (>90%), indicating limited scope for improvement. These high rates of adherence to therapy and complete treatment are often achieved through intentional clinical management programs that monitor patients’ treatment and adherence [43, 44]. Despite seeing a lack of overall change in adherence to treatment, changes in adherence may be different across subgroups, such as those with a high comorbidity burden, [45] although additional research is warranted to assess this.

This study has a number of limitations to consider. First, limited information was available on actual rebate amounts for individual payers, so public statements from manufacturers [18, 19] and data on discounts [26] were used to support our assumptions around list prices reducing to the approximate net prices and to add confidence to our interpretation of the findings. Nonetheless, as variation exists between payers on discounts provided, our findings may not be generalizable to all payers. Second, despite adjusting for plan types, we did not have details on formulary tiers, which may explain the differences observed in patterns of OOP cost distributions between PCSK9is and HCVs. For example, some plans may have placed both branded and authorized generic products on formulary tiers with similar cost-sharing requirements, which may explain the smaller changes in OOP costs for HCVs compared with PCSK9is [46, 47]. Lastly, because the database lacked detailed clinical information it was assumed that patients with at least two prescriptions of ledipasvir/sofosbuvir received a complete treatment regimen. In practice, however, some patients may have needed three prescriptions to complete the regimen. Despite these limitations, the large sample sizes and robust study design, including multiple covariate adjustments, are key strengths to the study. Future research is warranted if data around individual benefit designs become available and could further elucidate the flow of rebates from manufacturer to patients.

## Conclusions

List price reductions to that of net prices resulted in lower OOP costs, suggesting that future insurance benefit design reform that involves reduction of rebates and/or changes in benefit designs may also potentially result in lower OOP costs for patients; however, further transparency across the drug supply chain is needed to determine the extent to which additional savings could be realized.

## Supporting information

S1 Table List of NDC codes. (DOCX)

S2 Table Unadjusted outcomes. (DOCX)

S1 Fig Patient attrition for A) PCSK9is (evolocumab and alirocumab) and B) HCV medications (velpatasvir/sofosbuvir and ledipasvir/sofosbuvir).
(DOCX)

S2 Fig Estimated discounts for original versus lower priced versions. (DOCX)
