# Supplementary material for: Impact of list price changes on out-of-pocket costs and adherence in four high-rebate specialty drugs
Source: PLoS One. 2023 Jan 19;18(1):e0280570. doi: 10.1371/journal.pone.0280570 (PMC9851557; doi:10.1371/journal.pone.0280570)
Supplement: S1 Table — (DOCX) [file pone.0280570.s001.docx]

**Table S1. List of NDC codes**

| Evolocumab | Alirocumab | Velpatasvir/sofosbuvir | Ledipasvir/sofosbuvir |
| --- | --- | --- | --- |
| Original NDC | Original NDC | Brand | Brand |
| 55513-0750-01, 55513-0760-01, 55513-0760-02, 55513-0770-01 | 00024-5901-01, 00024-5901-02, 00024-5902-00, 00024-5902-01, 00024-5902-02, 00024-5903-01, 00024-5903-02, 00024-5904-01, 00024-5904-02 | 61958-2201-01 | 61958-1801-01, 61958-1803-01 |
| New NDC | New NDC | Authorized generic | Authorized generic |
| 72511-0750-01, 72511-0760-01, 72511-0760-02, 72511-0770-01 | 61755-0020-01, 61755-0020-02, 61755-0021-01, 61755-0021-02, 72733-5901-01, 72733-5901-02, 72733-5902-02 | 72626-2701-01 | 72626-2601-01 |

NDC, national drug code
