# Supplementary material for: Impact of list price changes on out-of-pocket costs and adherence in four high-rebate specialty drugs
Source: PLoS One. 2023 Jan 19;18(1):e0280570. doi: 10.1371/journal.pone.0280570 (PMC9851557; doi:10.1371/journal.pone.0280570)
Supplement: S2 Table — (DOCX) [file pone.0280570.s002.docx]

#### **Table S2. Unadjusted outcomes**

| **Outcome** | **PCSK9i** | | | | | | | | | |
| --- | --- | --- | --- | --- | --- | --- | --- | --- | --- | --- |
|  | **Evolocumab** | | | |  | **Alirocumab** | | | |  |
|  | **Original NDC** | | **New NDC** | |  | **Original NDC** | | **New NDC** | |  |
|  | **n** | **%** | **n** | **%** | ***p* value** | **n** | **%** | **n** | **%** | ***p* value** |
| Mean OOP costs per Rx, US$ | 268 | 290 | 128 | 139 | < 0.01 | 169 | 237 | 100 | 114 | < 0.01 |
| OOP costs per Rx without deductible, US$ | 161 | 187 | 73 | 80 | < 0.01 | 128 | 194 | 72 | 100 | < 0.01 |
| 6-month PDC | 0.83 | 0.21 | 0.85 | 0.19 | 0.33 | 0.84 | 0.22 | 0.84 | 0.21 | 0.23 |
| Days of follow-up | 471 | 85 | 448 | 85 | < 0.01 | 418 | 72 | 314 | 89 | < 0.01 |
| **Outcome** | **HCV medication** | | | | | | | | | |
|  | **Velpatasvir/sofosbuvir** | | | | | **Ledipasvir/sofosbuvir** | | | | |
|  | **Branded** | | **Generic** | |  | **Branded** | | **Generic** | |  |
|  | **n.** | **%** | **n** | **%** | ***p* value** | **n** | **%** | **n** | **%** | ***p* value** |
| Mean OOP costs per Rx (SD), $ | 751 | 1,622 | 570 | 1,057 | 0.94 | 868 | 2,117 | 810 | 1,522 | 0.41 |
| Mean OOP costs per Rx without deductible (SD), $ | 529 | 1,325 | 385 | 839 | 0.69 | 642 | 1,953 | 556 | 1,256 | 0.88 |
| Patients with at least three prescriptions | 1,640 | 91.6 | 1,037 | 89.3 | 0.04 | NA | NA | NA | NA | NA |
| Patients with at least two prescriptions | NA | NA | NA | NA | NA | 796 | 96.3 | 361 | 94.8 | 0.23 |

HCV, hepatitis C virus; NA, not applicable; NDC, national drug code; OOP, out of pocket; PCSK9i, proprotein convertase subtilisin/kexin type 9 inhibitor; PDC, proportion of days covered; Rx, prescription; SD, standard deviation.
