# Supplementary material for: Impact of list price changes on out-of-pocket costs and adherence in four high-rebate specialty drugs
Source: PLoS One. 2023 Jan 19;18(1):e0280570. doi: 10.1371/journal.pone.0280570 (PMC9851557; doi:10.1371/journal.pone.0280570)
Supplement: S1 Fig — Patient attrition for A) PCSK9is (evolocumab and alirocumab) and B) HCV medications (velpatasvir/sofosbuvir and ledipasvir/sofosbuvir). (DOCX) [file pone.0280570.s003.docx]

#### **Fig S1.** **Patient attrition for A) PCSK9is (evolocumab and alirocumab) and B) HCV medications (velpatasvir/sofosbuvir and ledipasvir/sofosbuvir)**


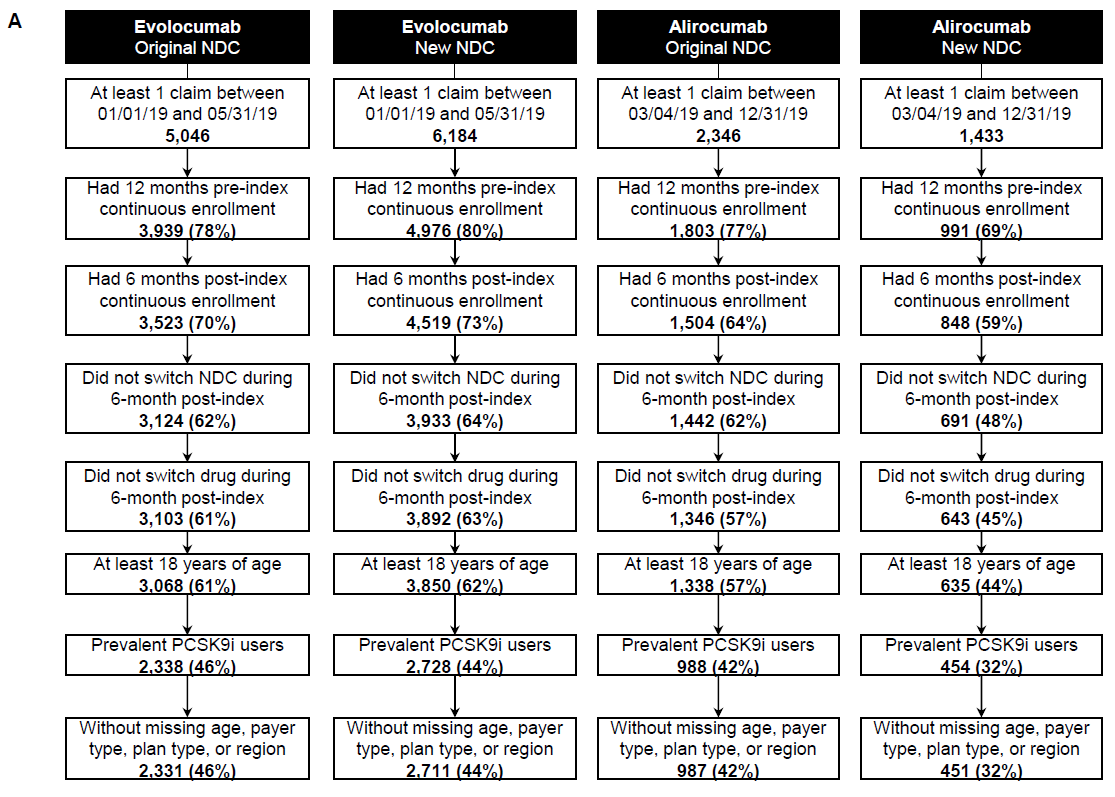


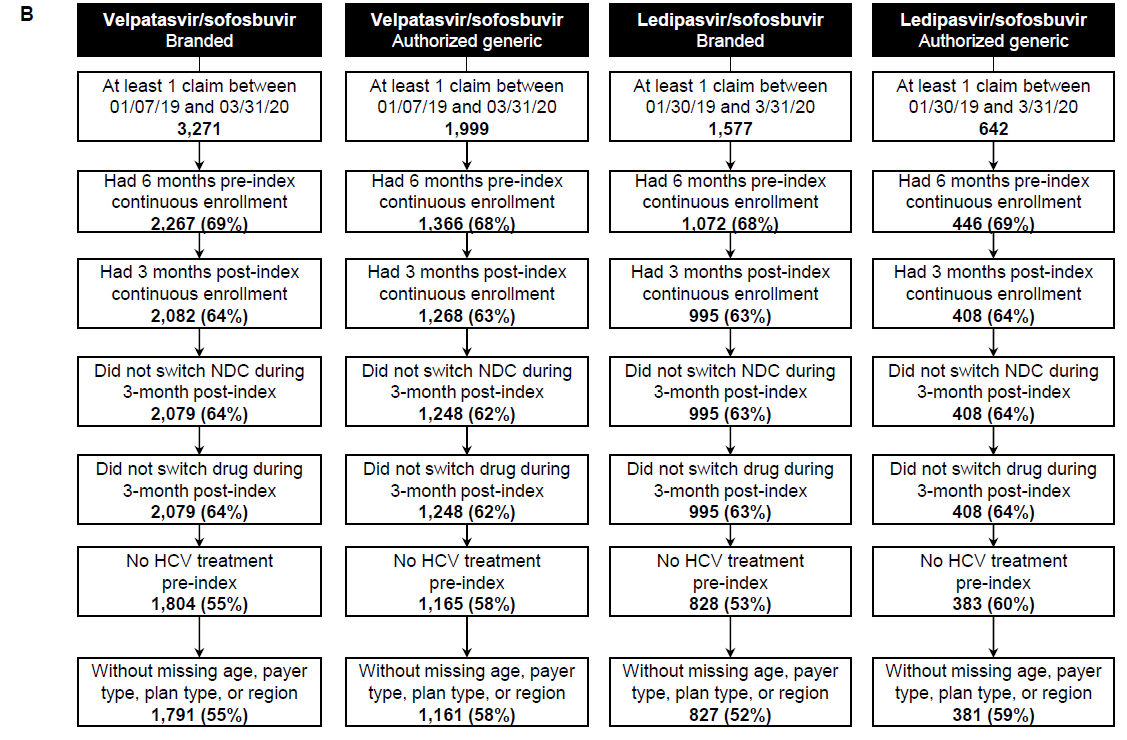


HCV, hepatitis C virus; NDC, national drug code; PCSK9i, proprotein convertase subtilisin/kexin type 9 inhibitor.
