# Supplementary material for: Impact of list price changes on out-of-pocket costs and adherence in four high-rebate specialty drugs
Source: PLoS One. 2023 Jan 19;18(1):e0280570. doi: 10.1371/journal.pone.0280570 (PMC9851557; doi:10.1371/journal.pone.0280570)
Supplement: S2 Fig — (DOCX) [file pone.0280570.s004.docx]

#### **Fig S2.** **Estimated discounts for (A) original version and lower priced versions of PCSK9s and (B) original (brand) versions compared to list prices of lower priced (authorized generic) versions of HCV medicines**

####

**A**

Discounts for the original version estimated using four quarter moving average for the quarter prior to list price reductions (evolocumab: Q4 2018, alirocumab: Q1 2019). Discounts for the lower priced version estimated using the four quarter moving average for the fourth quarter after the original versions removed from the market (evolocumab: Q4 2020, alirocumab: Q2 2022). Data estimated using the SSR Health LLC database.

**B**

Discounts and list prices based on course of therapy assuming 3 doses. Discounts for the original (brand) version estimated using four quarter moving average for the fourth quarter after the launch of the authorized generics (Q1 2020). Discount data estimated using the SSR Health LLC database. Discounts for authorized generics were not available, however the discounts of original (brand) versions were larger in comparison to the list prices of the authorized generics, thus indicating that any discounts would with the authorized generics would be less. List price data for authorized generics were estimated from Analysource pricing database.
